# CRE: a cost effective and rapid approach for PCR-mediated concatenation of *KRAS* and *EGFR* exons: Rapid way to detect *EGFR* and *KRAS* mutations

**DOI:** 10.12688/f1000research.6663.2

**Published:** 2016-03-08

**Authors:** Manoj P. Ramteke, Kuldeep J Patel, Mukul Godbole, Maulik Vyas, Kunal Karve, Anuradha Choughule, Kumar Prabhash, Amit Dutt

**Affiliations:** 1 Advanced Centre for Treatment, Research and Education in Cancer, Tata Memorial Centre, Navi Mumbai, Maharashtra, 410210, India; 2 Department of Medical Oncology, Tata Memorial Centre, Mumbai, Maharashtra, 400012, India

**Keywords:** EGFR and KRAS mutation, multiplex-PCR, concatenation of PCR products, Clinical diagnostics

## Abstract

Molecular diagnostics has changed the way lung cancer patients are treated worldwide. Of several different testing methods available, PCR followed by directed sequencing and amplification refractory mutation system (ARMS) are the two most commonly used diagnostic methods worldwide to detect mutations at *KRAS* exon 2 and *EGFR* kinase domain exons 18-21 in lung cancer. Compared to ARMS, the PCR followed by directed sequencing approach is relatively inexpensive but more cumbersome to perform. Moreover, with a limiting amount of genomic DNA from clinical formalin-fixed, paraffin-embedded (FFPE) specimens or fine biopsies of lung tumors, multiple rounds of PCR and sequencing reactions often get challenging. Here, we report a cost-effective single multiplex-PCR based method, CRE (for **C**o-amplification of five *K **R**AS* and ***E*** *GFR* exons), followed by concatenation of the PCR product as a single linear fragment for direct sequencing. CRE is a robust protocol that can be adapted for routine use in clinical diagnostics with reduced variability, cost and turnaround time requiring a minimal amount of template DNA extracted from FFPE or fresh frozen tumor samples. As a proof of principle, CRE is able to detect the activating *EGFR* L858R and T790M *EGFR* mutations in lung cancer cell line and primary tumors.

## Introduction

The growing significance of identifying *EGFR* and *KRAS* mutations in lung cancer using molecular diagnostic approaches underlines the emphasis on the use of personalized medical care by physicians to help design optimal therapeutic regimens ( Lynch *et al.*, 2004; Paez *et al.*, 2004; Pao *et al.*, 2004; Pao *et al.*, 2005a; Pao *et al.*, 2005b). While *EGFR* and *KRAS* mutations largely occur mutually exclusively in non-small cell lung cancer (NSCLC), and predict contrasting response rate to tyrosine-kinase inhibitors (TKI) ( Chougule *et al.*, 2013; Fukuoka *et al.*, 2011; Ihle *et al.*, 2012; Lynch *et al.*, 2004; Mao *et al.*, 2010; Mok *et al.*, 2009), some recent studies, including ours, suggest co-occurrence of *EGFR* and *KRAS* mutations in the same patients, albeit at low frequency ( Choughule *et al.*, 2014; Li *et al.*, 2014). While no direct evidence exists as yet, these studies may have implications for carrying out routine KRAS molecular testing along with EGFR mutations for precluding a patient with NSCLC from therapy with EGFR inhibitors, as approved for colorectal cancer ( Lievre *et al.*, 2006). Such information is especially important for lung cancer patients at an advanced-stage, who are not candidates for surgical intervention—wherein biopsy specimens obtained through fine-needle aspiration (FNA) may represent the only opportunity to obtain tissue material for diagnosis and molecular diagnostic analysis.

*EGFR* mutations in NSCLC are characterized by approximately 39 unique mutations present across exons 18-21. Of these, most common are activating mutations, which account for approximately 90% of all *EGFR* mutations and are closely related to the efficacy of EGFR-TKIs. These activating mutations include point mutations G719S, T790M, L858R, and L861Q in exons 18, 20 and 21 respectively and in-frame deletions/insertions in exon 19 ( Kosaka *et al.*, 2004). The most common mutations that result in an amino acid substitution at position 12 and 13 in *KRAS* are G12V and G13D ( Choughule *et al.*, 2014). Several screening and target based methods are currently in use for to infer the *EGFR* and *KRAS* hot spot mutations, viz; PCR-RFLP (Restriction fragment length polymorphism), Amplification Refractory Mutation System (ARMS), PCR-Invader, TaqMan PCR, allele specific qPCR, high resolution melting analysis and ultra-deep pyrosequencing, SNaPshot analysis and co-amplification at lower denaturation temperature (COLD)-PCR ( Angulo *et al.*, 2012; Borràs *et al.*, 2011; Ellison *et al.*, 2013; Santis *et al.*, 2011; van Eijk *et al.*, 2011; Zinsky *et al.*, 2010). Of these, direct sequencing is the most commonly used method worldwide ( Yatabe *et al.*, 2015). However, a typical PCR reaction that precedes the sequencing step to amplify 4 *EGFR* and 1 *KRAS* exon(s) essentially consists of five rounds of independent PCR requiring separate aliquots of genomic DNA template for each reaction, followed by ten rounds of sequencing reactions. With a limited amount of genomic DNA from clinical FFPE specimens or fine biopsies of lung tumors, multiple rounds of PCR and sequencing reactions can often be challenging to perform.

In-frame concatenation or assembly of individually amplified exons from genomic DNA to generate a coding fragment has been described in earlier research, wherein the total number of PCR reactions corresponds to the number of exons to be concatenated ( An *et al.*, 2007; Fedchenko *et al.*, 2013; Mitani *et al.*, 2004; Tuohy & Groden, 1998). Here, we describe a novel methodology to co-amplify all four *EGFR* exons 18-21 along with *KRAS* exon 2 in a single multiplex PCR followed by directional or ordered concatenation of the products in the form of a single linear fragment. This concatenated product can be used to detect mutations by direct sequencing, at a much reduced cost and duration, and with a much smaller amount of template.

## Materials and methods

### Samples

Genomic DNA was isolated from human NSCLC cell line NCI-H1975 and primary fresh frozen tumor tissue using QIAamp DNA blood mini kit (Qiagen). Genomic DNA from FFPE blocks was isolated using QIAamp DNA FFPE tissue kit (Qiagen) as per manufacturer’s instructions. DNA concentration was determined by absorbance at 280 nm (NanoDrop 2000, Thermo Scientific).

### Primer design

PCR primers were designed for *KRAS* exon 2 and *EGFR* exons 18-21. Supplementary Table S1 represents all the primers used for PCR amplifications. With the exception of the OAD176 and OAD152 primers, all internal primers contain an additional overhang of 15 nucleotides, such that the tail sequence of forward and reverse primers of two subsequent exons are complementary to each other to allow ordered and directional concatenation of *KRAS* and *EGFR* exons. The full length concatenated product of 915 bases was amplified using OAD176 and OAD152 primers.

### Multiplex PCR of *KRAS* exon 2 and *EGFR* exons 18-21

Multiplex PCR (50 µl per reaction) was carried out in a single tube by using multiplex PCR kit (Qiagen) containing either 10 ng of genomic DNA from the NSCLC cell line or fresh frozen primary tumor, or 50 ng of genomic DNA from FFPE blocks with 0.2 µM each of the five primer pairs using Applied Biosystems Veriti 96-Well Thermal Cycler. PCR was carried out with initial hot-start denaturation at 95°C for 15 min, followed by 35 cycle of denaturation at 94°C for 30 seconds, annealing at 57°C for 90 seconds, polymerization at 72°C for 60 seconds, and final incubation for 30 min at 60°C. The multiplex PCR products were analyzed by agarose gel electrophoresis.

### Concatenation of exons and sequencing analysis

For concatenation of *KRAS* exon 2 and *EGFR* exons 18-21, 2 µl of multiplex PCR product was used as template in a 50 µl PCR reaction containing 0.2 µM of each OAD176 and OAD152 primers. PCR was carried out in a Verity thermal cycler (Applied Biosystems) with an initial hot-start denaturation at 95°C for 15 min, followed by 35 cycle of denaturation at 94°C for 30 seconds, annealing at 57°C for 90 seconds, polymerization at 72°C for 60 seconds, and final incubation for 30 min at 60°C. Concatenated PCR product was analyzed by agarose gel electrophoresis. Sequencing of concatenated PCR products were performed by Sanger sequencing. Sequences were analyzed using Mutation Surveyor software V4.0.9 ( Minton *et al.*, 2011).

## Results

**Figure 1.**
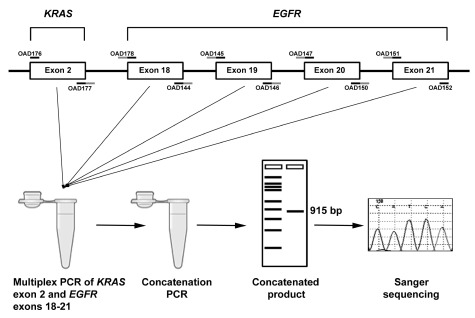
Schematic representation of CRE: Concatenation of *K RAS* and *EGFR* exons. The flowchart represents the workflow for CRE methodology. *KRAS* and *EGFR* primers are shown along with complementary tail overhangs that prime with consecutive exons in an ordered manner. 2 µl PCR products, amplified with a cocktail of primers, as shown and described in Supplementary Table S1, for *KRAS* and *EGFR* exons in a single multiplex reaction is transferred to a fresh tube and concatenated in a separate reaction using OAD 176 and OAD 152 primers. The concatenated product obtained is a single product of 915 bp with all individual exons amplified from multiplex PCR ligated together in an ordered manner as a single fragment. 2x sequencing using the forward primer OAD 176 and reverse primer OAD 152 of the concatenated product is adequate to scan the mutation status across all the *KRAS* and *EGFR* exons.

CRE ( **C**o-amplification of *K **R**AS* and **E**GFR) exons is a cost-effective multiplex-PCR based method followed by concatenation of the PCR product as a single fragment for direct sequencing ( Figure 1). It is a robust methodology to determine the mutation status of *KRAS* and *EGFR* with reduced variability, cost and turnaround time, requiring a minimal amount of template DNA extracted from FFPE or fresh frozen tumor samples.

### CRE-based *KRAS*- *EGFR* concatenation from fresh frozen primary tumors and tumor-derived cell lines

**Figure 2.**
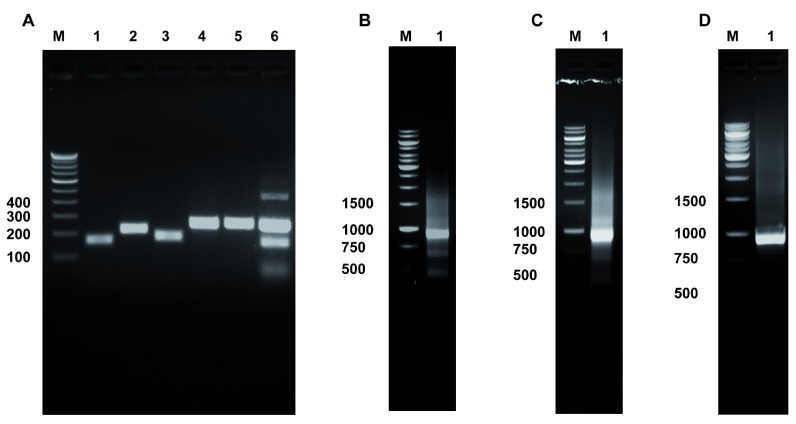
Multiplex PCR amplification and concatenation of *KRAS* and *EGFR* exons generates CRE product. **Panel A**. PCR amplification of *KRAS* and *EGFR* exons using NCI-H1975 genomic DNA: Lane 1, *KRAS* exon 2 (151 bp) amplified with OAD176 and OAD177; Lane 2, *EGFR* exon 18 (209 bp) amplified with OAD 178 and OAD 144; Lane 3, *EGFR* exon 19 (178 bp) amplified with OAD 145 and OAD 146; Lane 4, *EGFR* exon 20 (246 bp) amplified with OAD 147 and OAD 150; Lane 5, *EGFR* exon 21 (251 bp) amplified with OAD 151 and OAD 152; Lane 6, Multiplex PCR of *KRAS* exon 2 and *EGFR* exons 18-21 with cocktail of primers used in Lanes 1–5. Concatenated *KRAS* and *EGFR* (CRE) product of ~915 bp amplified with OAD 176 and OAD 152 using multiplex PCR product as template derived from NCI-H1975 genomic DNA (shown in **Panel B**, Lane 2); derived from fresh frozen primary tumor genomic DNA (shown in **Panel C**, Lane 2); using tumor genomic DNA extracted from FFPE block (shown in **Panel D,** Lane 2).

**Figure 3.**
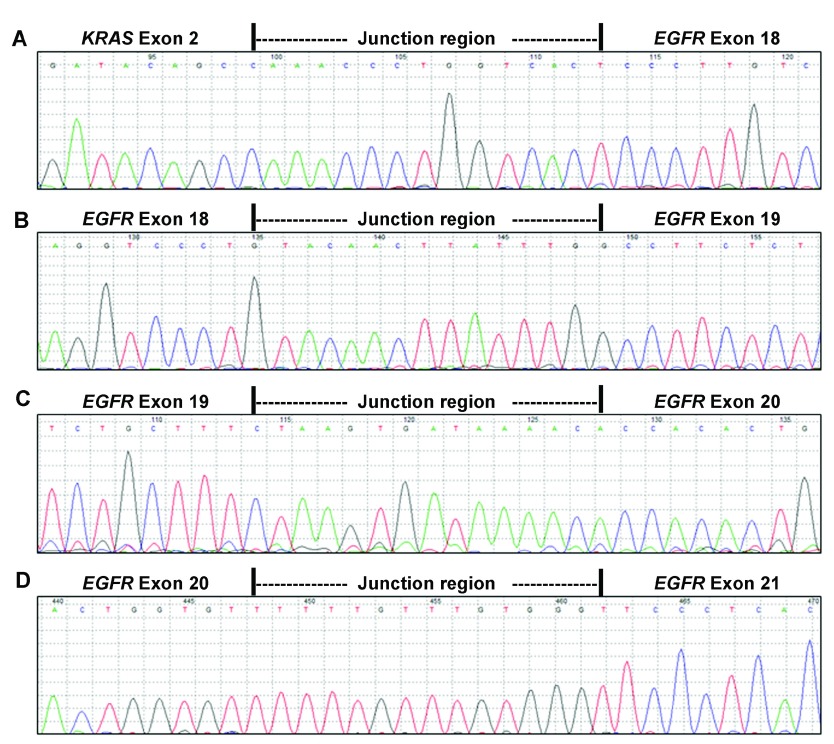
Full length sequencing of the CRE product. Reverse complements of the forward sequencing reads of the 915 bp *KRAS*- *EGFR* concatenated product are displayed as generated by Mutation Surveyor V4.0.9. **Panel A** displays 15 nucleotide junction region flanked by *KRAS* exon 2 and *EGFR* exon 18 sequence; **Panel B** displays 15 nucleotide junction region flanked by *EGFR* exons 18 and 19; **Panel C** displays 15 nucleotide junction region flanked by *EGFR* exons 19 and 20; and displays 15 nucleotide junction region flanked by *EGFR* exons 20 and 21 is shown in **Panel D**.

**Figure S1.**
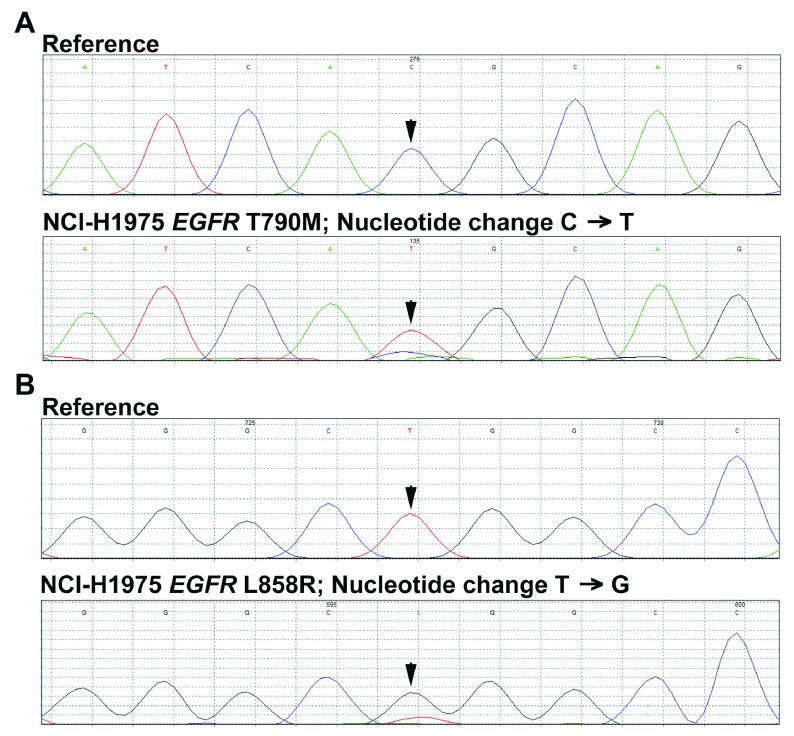
Detection of *EGFR* T790M and L858R mutations from NCI-H1975 CRE product. Reverse complements of the forward sequencing reads of the 915 bp CRE product using genomic DNA extracted from NCI-H1975 cells are displayed as generated by Mutation Surveyor. **Panel A**: The arrow indicates expected location of the wild-type and T790M mutant allele peak. **Panel B**: The arrow indicates expected location of the wild-type and L858R mutant allele peak.

Following CRE-based multiplex PCR of *KRAS* exon 2 and *EGFR* exons 18-21 with overlapping PCR bands ( Figure 2A, lane 6), concatenation of the PCR product was performed with OAD176 and OAD152 primers using genomic DNA extracted from NCI-H1975 cells, a non-small-cell lung adenocarcinoma cell line. Concatenation PCR resulted in the enrichment of a concatenated product of about 915 base pairs ( Figure 2B). This concatenated, gel purified PCR product of 915 base pair was used for Sanger sequencing. Sequencing analysis of the concatenated PCR product confirmed concatenation as a single fragment ( Figure 3) along with the presence of *EGFR* T790M and L585R mutations in NCI-H1975 cells ( Supplementary Figure S1). A similar concatenation of a 915 bp single fragment was performed with genomic DNA extracted from fresh frozen tumor cells ( Figure 2C).

Raw gel electrophoresis images for Figure 2: Multiplex PCR amplification and concatenation of *KRAS* and *EGFR* exons generates CRE product Zip file contains 4 files: Raw image for Figure 2A, Raw image for Figure 2B, Raw image for Figure 2C, Raw image for Figure 2D.
Panel A. PCR amplification of *KRAS* and *EGFR* exons using NCI-H1975 genomic DNA: Lane 1, *KRAS* exon 2 (151 bp) amplified with OAD176 and OAD177; Lane 2, *EGFR* exon 18 (209 bp) amplified with OAD 178 and OAD 144; Lane 3, *EGFR* exon 19 (178 bp) amplified with OAD 145 and OAD 146; Lane 4, *EGFR* exon 20 (246 bp) amplified with OAD 147 and OAD 150; Lane 5, *EGFR* exon 21 (251 bp) amplified with OAD 151 and OAD 152; Lane 6, Multiplex PCR of *KRAS* exon 2 and *EGFR* exons 18–21 with cocktail of primers used in Lanes 1–5.
Concatenated *KRAS* and *EGFR* (CRE) product of ~915 bp amplified with OAD 176 and OAD 152 using multiplex PCR product as template derived from NCI-H1975 genomic DNA (shown in **Panel B**, Lane 2); derived from fresh frozen primary tumor genomic DNA (shown in **Panel C**, Lane 2); using tumor genomic DNA extracted from FFPE block (shown in **Panel D,** Lane 2) ( Ramteke *et al.*, 2015a). Click here for additional data file. Copyright: © 2016 Ramteke MP et al. 2016 Data associated with the article are available under the terms of the Creative Commons Zero "No rights reserved" data waiver (CC0 1.0 Public domain dedication).

Sequencing traces for Figure 3: Full length sequencing of the CRE product Zip file contains 4 files: Sequencing trace for Figure 3A .ab1, Sequencing trace for Figure 3B .ab1, Sequencing trace for Figure 3C .ab1 and Sequencing trace for Figure 3D .ab1.
Reverse complements of the forward sequencing reads of the 915 bp *KRAS*- *EGFR* concatenated product are displayed as generated by Mutation Surveyor V4.0.9. Panel A displays 15 nucleotide junction region flanked by *KRAS* exon 2 and *EGFR* exon 18 sequence; Panel B displays 15 nucleotide junction region flanked by *EGFR* exons 18 and 19; Panel C displays 15 nucleotide junction region flanked by *EGFR* exons 19 and 20; and displays 15 nucleotide junction region flanked by *EGFR* exons 20 and 21 is shown in Panel D ( Ramteke *et al.*, 2015b). Click here for additional data file. Copyright: © 2016 Ramteke MP et al. 2016 Data associated with the article are available under the terms of the Creative Commons Zero "No rights reserved" data waiver (CC0 1.0 Public domain dedication).

### CRE-based *KRAS*- *EGFR* concatenation from paraffin-embedded clinical cancer specimens

**Figure S2.**
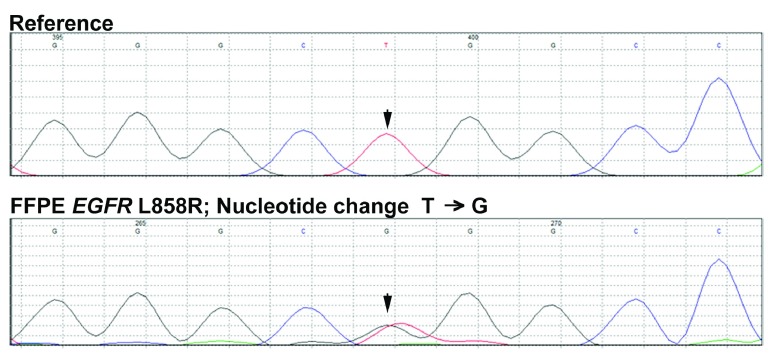
Detection of *EGFR* L858R mutation in a CRE product derived from FFPE primary tumor sample. Reverse complements of the forward sequencing reads of the 915 bp CRE product using genomic DNA extracted from FFPE primary tumor are displayed are displayed as generated by Mutation Surveyor. The arrow indicates expected location of the wild-type and L858R mutant allele peak.

The amount of genomic DNA obtained from FFPE tissue is always limiting and thus there is a substantial need to develop a technique with a limited amount of starting DNA as a template for mutation detection. CRE demonstrates the ability to co-amplify all five exons ( *KRAS* exon 2 and *EGFR* exon 18-21) in a single multiplex PCR reaction with a limited amount of starting template DNA followed by the enrichment of concatenated product ( Figure 2D) by concatenation PCR using first multiplex PCR product as a template. The concatenated product confirmed *EGFR* L858R mutation in the FFPE tissues ( Supplementary Figure S2), as reported earlier ( Choughule *et al.*, 2014). Thus our CRE method can be routinely used for the mutational analysis of *KRAS* and *EGFR* genes.

## Discussion

CRE is a novel, simple and effective strategy to concatenate multiple amplicons obtained from a multiplex PCR, using primers with overlapping complementary overhangs. Compared to ARMS, and other genotyping technologies, CRE is relatively inexpensive with faster turnaround time involving lesser amount of template genomic DNA.

Using CRE, *in vitro* tandem reconstitution of *KRAS* exon 2 with *EGFR* exons 18-21 can be effectively performed to generate a concatenated single PCR product of 915 bp, as a template for sequencing. Most commercially-available allele-specific and genotyping technologies are restricted by their ability to probe only for eight out of the approximately 39 known commonly occurring *EGFR* and *KRAS* activating mutations. However, growing clinical data on the less common mutations are now emerging to fully inform their predictable outcomes on EGFR TKIs ( Lohinai *et al.*, 2015; Yang *et al.*, 2012). Currently available methodologies, if extended to genotype all known 39 mutations would not only be cost-prohibitive but challenging to perform due to a limiting amount of template genomic DNA available from clinical cancer specimens that are mostly available in the form of formalin-fixed, paraffin-embedded (FFPE) tissue. While a directed sequencing approach –classical or next-generation sequencing (NGS) -based—can determine a whole spectrum of rare and co-occurring mutations in an individual, the question of template genomic DNA availability still remains. CRE circumvents the issue of a limiting amount of template genomic DNA with increased affordability by multiplexing PCR for all exons to a single reaction and concatenating the PCR product as a single fragment, thereby further reducing the cost of multiple sequencing reactions.

In this era of genome sequencing, applicability of the CRE strategy could be of immense significance to effectively reduce the cost and turnaround time taken to determine the mutational status across the whole *KRAS* exon 2 and *EGFR* kinase domain exons. As the limitation of the CRE strategy is defined by the sensitivity and resolution of the sequencing methodology adopted, concatenated *EGFR* and *KRAS* PCR products from multiple individuals—each tagged with unique bar code sequence—can be pooled and deep-sequenced using a NGS platform. The CRE strategy described here can reduce the labor and cost of performing individual PCR for all exons for each patient and effectively circumvent the noise due to variation in normalization for equimolar pooling of exons within the same sample at a resolution of single base. Additionally, the current version of CRE is limited by exclusion of fewer number of exons of *EGFR* and *KRAS*. Inclusion of known extracellular *EGFR* and *KRAS* exon 3 codon 61 mutation may help to immediately expand the scope of its application to other cancers, such as glioblastoma.

## Data availability

The data referenced by this article are under copyright with the following copyright statement: Copyright: © 2016 Ramteke MP et al.

Data associated with the article are available under the terms of the Creative Commons Zero "No rights reserved" data waiver (CC0 1.0 Public domain dedication). 

*F1000Research*: Dataset 1. Raw gel electrophoresis images for Figure 2: Multiplex PCR amplification and concatenation of *KRAS* and *EGFR* exons generates CRE product, 10.5256/f1000research.6663.d50236

*F1000Research*: Dataset 2. Sequencing traces for Figure 3: Full length sequencing of the CRE product, 10.5256/f1000research.6663.d50237

*F1000Research*: Dataset 3. Sequencing traces for Figure S1: Detection of *EGFR* T790M and L858R mutations from NCI-H1975 CRE product, 10.5256/f1000research.6663.d50238

*F1000Research*: Dataset 4. Sequencing trace for Figure S2: Detection of *EGFR* L858R mutation in a CRE product derived from FFPE primary tumor sample, 10.5256/f1000research.6663.d50239

## Figures and Tables

**Supplementary Table S1.** Sequences with underline denote priming region of primer. Sequences in italics indicate extra 15 nucleotide tail sequences (junction region). Sequences in bold denotes complementary region between reverse primer of one exon with forward primers of successive exon. 5′ and 3′ represents forward and reverse primer respectively.

Primer	Primer information	Amplicon size (bp)	Sequences
OAD176	5′ *KRAS* exon 2	151	C C TTATGTGTGACAT GTTCTAATATAGTCA C
OAD177	3′ *KRAS* exon 2		**ACACAGAGACAAGGG** *AGTGACCAGGGTTTG* GCTGTATCGTCAA GGC AC
OAD178	5′ *EGFR* exon 18	209	*CAAACCCTGGTCACT* **CCCTT GT CTCTGT GT**TCTTGTC C C C CC C AG
OAD144	3′ *EGFR* exon 18		**CTATGACAGAGAGAGAAGG** *CCAAATAAGTTGTAC* AGGGACC TTACC TTA
OAD145	5′ *EGFR* exon 19	178	*GTACAACTTATTTGG* **CCTTCTCTCTCTGTCATAG**GGACTCTGGAT
OAD146	3′ *EGFR* exon 19		**GGCACGTCAGTGTGG** *TGTTTTATCACTTAG* AAAGC AGA AAC TCAC
OAD147	5′ *EGFR* exon 20	246	*CTAAGTGATAAAACA* **CCACACT GAC GT GCC**TCTC C C TCC C TCC AG
OAD150	3′ *EGFR* exon 20		**CCCTGCTGTGAGGGA** *ACCCACAAACAAAAA*ACACCAGTTGAGCAG
OAD151	5′ *EGFR* exon 21	251	*TTTTTGTTTGTGGGT* **TCCCTCACA GCA GG G**TCTTC TCTGTTTCA G
OAD152	3′ *EGFR* exon 21		TGGTC C C TGGTGTC A GGAA

## Supplementary materials

Sequencing traces for Figure S1: Detection of *EGFR* T790M and L858R mutations from NCI-H1975 CRE product Zip file contains 2 files: Sequencing trace for Figure S1B .ab1 and Sequencing trace for Figure S1A .ab1 Reverse complements of the forward sequencing reads of the 915 bp CRE product using genomic DNA extracted from NCI-H1975 cells are displayed as generated by Mutation Surveyor. Panel A: The arrow indicates expected location of the wild-type and T790M mutant allele peak. Panel B: The arrow indicates expected location of the wild-type and L858R mutant allele peak ( Ramteke *et al.*, 2015c). Copyright: © 2016 Ramteke MP et al.2016Data associated with the article are available under the terms of the Creative Commons Zero "No rights reserved" data waiver (CC0 1.0 Public domain dedication).

Sequencing trace for Figure S2: Detection of *EGFR* L858R mutation in a CRE product derived from FFPE primary tumor sample Reverse complements of the forward sequencing reads of the 915 bp CRE product using genomic DNA extracted from FFPE primary tumor are displayed are displayed as generated by Mutation Surveyor. The arrow indicates expected location of the wild-type and L858R mutant allele peak ( Ramteke *et al.*, 2015d). Copyright: © 2016 Ramteke MP et al.2016Data associated with the article are available under the terms of the Creative Commons Zero "No rights reserved" data waiver (CC0 1.0 Public domain dedication).
