# Supplementary material for: CRE: a cost effective and rapid approach for PCR-mediated concatenation of KRAS and EGFR exons: Rapid way to detect EGFR and KRAS mutations
Source: F1000Res. 2016 Mar 8;4:160. Originally published 2015 Jun 23. [Version 2] doi: 10.12688/f1000research.6663.2 (PMC4830212; doi:10.12688/f1000research.6663.2)
Supplement: Peer review history

# Peer review: Referee response for version 2

**DOI:** 10.5256/f1000research.8838.r9437

**Published:** 2016-04-05

**Authors:** Chandan Kumar

**Affiliations:** 1 Department of Pathology, University of Michigan, Ann Arbor, MI, USA

The authors have provided satisfactory responses to all the concerns. Within the limits of sensitivity of Sanger sequencing, and currently limited to the the exons examined, the relatively simple, cost effective assay system presented here is a practical solution to an important clinical question in resource limited environment.

I have read this submission. I believe that I have an appropriate level of expertise to confirm that it is of an acceptable scientific standard.

# Peer review: Referee response for version 1

**DOI:** 10.5256/f1000research.7157.r9740

**Published:** 2016-02-24

**Authors:** Bob T. Li

**Affiliations:** 1 Thoracic Oncology Service, Division of Solid Tumor Oncology, Department of Medicine, Memorial Sloan Kettering Cancer Center, New York, NY, USA

In the age of precision medicine with an expanding number of oncogenic drivers in lung cancers that may be treated with targeted agents, multiplexed genomic testing is increasingly important in clinical practice. The study by Ramteke et al. describes a rapid and relatively inexpensive multiplexed test for EGFR and KRAS mutations. The methods are well described and the test is of clinical relevance, particularly in settings with limited resources and without access to tumor next generation sequencing. I recommend making the following minor revisions:

1. In the introduction, it is incorrect to suggest that the reason for KRAS testing in lung cancers is to preclude patients from EGFR inhibitors. The rationale for EGFR inhibitors in lung cancers is very different to that of colorectal cancers, as activating EGFR mutations in lung cancers predict response to EGFR TKIs. However, it is still important to test all lung cancers for KRAS mutations as it is a common oncogenic driver occuring in over 25% of lung adenocarcinomas. Being a driver KRAS is highly unlikely to co-exist with other actionable drivers, therefore once KRAS is found one could justify that further genomic testing for other drivers is not necessary, especially in a resource limited setting.
2. It should be acknowledged that the authors' CRE method will not capture all KRAS mutations, especially KRAS mutations in exon 3 codon 61. However, the ability to capture the majority of KRAS and EGFR mutations in one single inexpensive test is still of value for patients with lung cancers.

I have read this submission. I believe that I have an appropriate level of expertise to confirm that it is of an acceptable scientific standard.

# Response

**Published:** 2016-02-24

**Authors:** Amit Dutt

**Affiliations:** ACTREC, Tata Memorial Center,, India

We sincerely thank the reviewer for the elaborate and detailed constructive review. In particular, we are grateful to the reviewer for describing the study as *“a well conducted proof of principle report”. *We hope the reviewer will find the improved version of the manuscript acceptable, without reservation. Our response to specific concerns are as follows *-*

**Referee’s comment 1:** *Introduction* *, “These studies have direct implications for carrying out routine KRAS molecular testing along with EGFR mutations for precluding a patient with NSCLC from therapy with EGFR inhibitors, as approved for colorectal cancer (Lievre et al., 2006).” Can the authors cite any reference wherein NSCLC patients are precluded from EGFR inhibitor therapy if they harbor KRAS mutations. The reference cited here is specific to colorectal cancer.*

**Author’s response:** *EGFR* and *KRAS* mutations occur mutually exclusive in NSCLC, which suggests functional redundancy, however they predict contrasting response rates to tyrosine-kinase inhibitors (TKI). While *EGFR* mutation predicts longer progression-free survival rate, adverse prognosis is associated with patients harboring *KRAS* mutations. Thus, the recently reported co-occurrence of *KRAS* and *EGFR* activating mutations in 30 of 5125 patients, along with our study of co-occurrence of *KRAS* and *EGFR* activating mutations in 3 of 86 patients, raises questions about the relative value of *EGFR* and *KRAS* mutation status as predictors of outcome in NSCLC. As the reviewer may agree these studies may have obvious implications for routine *KRAS* testing in this disease, potentially precluding EGFR TKI therapy from some patients, similar to current practice in colorectal cancer. However, their direct mention in NSCLC is speculative.

Thus, in principle, we fully agree with the reviewer that no direct evidence exists to preclude EGFR inhibitor therapy among patients co-harboring *EGFR *and *KRAS* mutation. In accordance with the reviewer’s suggestion we have revised the text to reflect the speculative implication of our methodology in NSCLC. Our modified text reads as follows:

“….While no evidence exists as yet, these studies may have implications for carrying out routine KRAS molecular testing along with EGFR mutations for precluding a patient with NSCLC from therapy with EGFR inhibitors, as approved for colorectal cancer (Lievre et al., 2006)….”

**Referee’s comment 2:** *Introduction* *, “Of these direct sequencing is the most commonly used method worldwide”. Is this a personal opinion or there is a reference to support this. Should be cited.*

**Author’s response: **We thank the reviewer for pointing the omission. A relevant citation has been added. However, as our citation in manuscript is likely to be incomplete to summarize the field, some additional studies are mentioned below:

Yatabe, Yasushi, et al. "EGFR Mutation Testing Practices within the Asia Pacific Region: Results of a Multicenter Diagnostic Survey." *Journal of Thoracic Oncology* 10.3 (2015): 438;

Angulo, Bárbara, et al. "A comparison of EGFR mutation testing methods in lung carcinoma: direct sequencing, real-time PCR and immunohistochemistry." *PLoS One* 7.8 (2012): e43842;

Ellison, Gillian, et al. "EGFR mutation testing in lung cancer: a review of available methods and their use for analysis of tumour tissue and cytology samples." *Journal of clinical pathology* 66.2 (2013): 79-89;

Cappuzzo, Federico. "Methods for EGFR Mutation Testing." *Guide to Targeted Therapies: EGFR mutations in NSCLC*. Springer International Publishing, 2014. 19-24.

**Referee’s comment 3:** *Introduction* *, “concatenation or assembly of individually amplified exons from genomic DNA to generate a cDNA fragment has been described in earlier research”. cDNA specifically refers to complementary DNA derived from RNA through reverse transcriptase. Genomic PCR cannot be said to be used to generate cDNA fragment. I suspect this erroneous phrasing is picked up from a previous reference, but its probably good to not perpetuate the error.*

**Author’s response: **We agree and thank the reviewer for bringing it our attention. Our modified text reads as follows:

“….concatenation or assembly of individually amplified exons from genomic DNA to generate a coding fragment has been described in earlier research…”.

**Referee’s comment 4:** *Introduction* *, “Here, we describe a novel methodology to co-amplify all four EGFR exons 18–21 along with KRAS exon 2 in a single multiplex PCR”. It's more like a novel application of a well described methodology supported by several previous references. The novelty, albeit rather incremental, is in combining exons from two different genes, using previously described approach. Should be stated as such.*

**Author’s response: **As suggested by the reviewer, we have dropped the term “novel”. Our modified text reads as follows:

“….Here, we describe a methodology to co-amplify all four *EGFR* exons 18–21 along with *KRAS* exon 2 in a single multiplex PCR….”

**Referee’s comment 5:** *The big appeal of the study is that it affords use of a minimal amount of FFPE sample. Please specify the amount of FFPE material used and yield of DNA to convey an idea of how little/much sample is needed to carry out this analysis.*

**Author’s response: **As mentioned in the methodology section subtitled, “Multiplex PCR of *KRAS *exon 2 and *EGFR* exons 18-21”, multiplex PCR (50µl per reaction) was carried out in a single tube by using multiplex PCR kit (Qiagen) containing either 10 ng of genomic DNA from the NSCLC cell line or fresh frozen primary tumor, or 50 ng of genomic DNA from FFPE blocks. Furthermore, as mentioned under the methodology section subtitled, “Concatenation of exons and sequencing analysis”, 2 µl of multiplex PCR product was used as template in a 50 µl PCR reaction for concatenation.

**Referee’s comment 6:** *The important consideration of sensitivity has not been addressed. This could be easily tested by assaying a serial dilution of known mutated cell line/FFPE DNA spiked in a wild-type background sample. This will add value to the study.*

**Author’s response: **As mentioned under the last paragraph of the discussion section, “….As the limitation of the CRE strategy is defined by the sensitivity and resolution of the sequencing methodology adopted…” – which in this study has been Sanger Sequencing, but could significantly vary if advanced contemporary sequencing methodologies are adopted. However, as the sensitivity of PCR followed by directed Sanger Sequencing is well established from FFPE samples and mutated cell line, we humbly differ from the reviewer that admixture experiment would add additional information.

**Referee’s comment 7:** *Addition of KRAS codon 61 should be considered as well. Or the difficulty in scaling up should be discussed. How difficult is it to add additional exons.*

**Author’s response:** *KRAS* codon 12 is mutated at a frequency of 25-50% in Caucasian population and 5-15% among East Asians. In a recent study we reported 18.6% *KRAS* codon 12 among Indian population (n=86)-- (Choughule *et al.*, 2014). Given that *KRAS* codon 61 mutation exist at frequency < 1%; and, that none were found in our study in 86 patients, we decided to not include *KRAS* codon 61 mutation in this study to only present the proof of principle of the CRE methodology. However, we do agree with the reviewer about the significance of *KRAS* codon 61 mutation, and do hope to include it along with other known activating mutations in NSCLC in an improved version of CRE.

However, to reflect the pertinent suggestion made by the reviewer we have modified our discussion to read as follows:

 “… Additionally, the current version of CRE is limited by exclusion of fewer number of exons of *EGFR* and *KRAS*. Inclusion of known extracellular *EGFR *and *KRAS* exon 3 codon 61 mutation may help to immediately expand the scope of its application to other cancers, such as glioblastoma.”

**Referee’s comment 8:** *The concatenated PCR product may be amenable to Pyrosequencing to improve sensitivity of detection (particularly in case of low tumor content, low clonality of mutations as is expected in case of dynamically evolving tumors). This should be attempted/ discussed.*

**Author’s response: ** We fully agree with the reviewer’s insights that CRE can be utilized at high throughput mode to determine complete spectrum of *EGFR* and *KRAS *mutations using targeted next generation sequencing. Consistent with the reviewer’s suggestion the last paragraph of the discussion section, “… the limitation of the CRE strategy is defined by the sensitivity and resolution of the sequencing methodology adopted, concatenated *EGFR* and *KRAS* PCR products from multiple individuals—each tagged with unique bar code sequence—can be pooled and deep-sequenced using a NGS platform. The CRE strategy described here can reduce the labor and cost of performing individual PCR for all exons for each patient and effectively circumvent the noise due to variation in normalization for equimolar pooling of exons within the same sample at a resolution of single base.”

**Referee’s comment 9:** *A direct comparison with the standard technique(s) currently used to test these mutations- in terms of amount of starting material needed, sensitivity of detection, time, and cost will help the argument of the new approach as a superior option.*

**Author’s response: ** As detailed in the manuscript, this proof of principle study introduces CRE as a methodology involving single multiplex-PCR followed by concatenation of the PCR product as one linear fragment for direct sequencing, as opposed to 5 rounds of PCR reaction followed by 10 rounds of sequencing reactions. A systematic comparative analysis is currently underway at our center using clinical cancer specimens for CRE compared to Sanger sequencing based methodology; SNaPShot PCR; Cobas system; Mass spec genotyping on a larger cohort sample, beyond the scope of this manuscript. Hence, we express our inability to include analysis from this ongoing study at this early on stage.

# Response

**Published:** 2016-02-24

**Authors:** Amit Dutt

**Affiliations:** ACTREC, Tata Memorial Center,, India

We sincerely thank reviewer for approving our submission. We are particularly grateful to the reviewer for describing the study as, *“The methods are well described and the test is of clinical relevance, particularly in settings with limited resources and without access to tumor next generation sequencing”. * The suggestions made by the reviewers have contributed to an improved version of the manuscript. Specifically, we have, in the revised version:

**Re** **feree’s comment 1:** *In the introduction, it is incorrect to suggest that the reason for KRAS testing in lung cancers is to preclude patients from EGFR inhibitors. The rationale for EGFR inhibitors in lung cancers is very different to that of colorectal cancers, as activating EGFR mutations in lung cancers predict response to EGFR TKIs. However, it is still important to test all lung cancers for KRAS mutations as it is a common oncogenic driver occuring in over 25% of lung adenocarcinomas. Being a driver KRAS is highly unlikely to co-exist with other actionable drivers, therefore once KRAS is found one could justify that further genomic testing for other drivers is not necessary, especially in a resource limited setting.*

**Author’s response: ** As described in or response to Reviewer 1’s first comment, we agree we with the reviewer that no direct evidence exists to preclude EGFR inhibitor therapy among patients co-harboring *EGFR *and *KRAS* mutation. In accordance with the reviewer’s suggestion we have revised the text to reflect the speculative implication of our methodology in NSCLC. Our modified text reads as follows:

“….While no evidence exists as yet, these studies may have implications for carrying out routine KRAS molecular testing along with EGFR mutations for precluding a patient with NSCLC from therapy with EGFR inhibitors, as approved for colorectal cancer (Lievre et al., 2006)….”

**Re** **feree’s comment 2:** *It should be acknowledged that the authors' CRE method will not capture all KRAS mutations, especially KRAS mutations in exon 3 codon 61. However, the ability to capture the majority of KRAS and EGFR mutations in one single inexpensive test is still of value for patients with lung cancers.*

**Author’s response: ** As described in our response to Reviewer 1’s comment 7, we agree with the reviewer about the significance of *KRAS* codon 61 mutation, and do hope to include it along with other known activating mutations in NSCLC. However, to reflect the pertinent suggestion made by the reviewer we have modified our discussion to read as follows:

 “… Additionally, the current version of CRE is limited by exclusion of fewer number of exons of *EGFR* and *KRAS*. Inclusion of known extracellular *EGFR *and *KRAS* exon 3 codon 61 mutation may help to immediately expand the scope of its application to other cancers, such as glioblastoma.”

# Peer review: Referee response for version 1

**DOI:** 10.5256/f1000research.7157.r9749

**Published:** 2015-07-31

**Authors:** Chandan Kumar

**Affiliations:** 1 Department of Pathology, University of Michigan, Ann Arbor, MI, USA

This is a well conducted proof of principle report that uses the approach of concatenated PCR of exons from genomic DNA to combine exons of two different, clinically relevant genes KRAS and EGFR, to address a clinically relevant question in a resource limiting setting. The assay design and data are provided in sufficient details that any researchers may be able to attempt to carry out similar analyses.

There are some comments and suggestions to improve the quality of the manuscript or follow up analyses:

1.  
**Introduction**, *“These studies have direct implications for carrying out routine KRAS molecular testing along with EGFR mutations for precluding a patient with NSCLC from therapy with EGFR inhibitors, as approved for colorectal cancer (Lievre et al., 2006).”*
  Can the authors cite any reference wherein NSCLC patients are precluded from EGFR inhibitor therapy if they harbor KRAS mutations. The reference cited here is specific to colorectal cancer.
2. **Introduction**, *“Of these direct sequencing is the most commonly used method worldwide”.*
  Is this a personal opinion or there is a reference to support this. Should be cited.
3. **Introduction**, *“concatenation or assembly of individually amplified exons from genomic DNA to generate a cDNA fragment has been described in earlier research”.*
  cDNA specifically refers to complementary DNA derived from RNA through reverse transcriptase. Genomic PCR cannot be said to be used to generate cDNA fragment. I suspect this erroneous phrasing is picked up from a previous reference, but its probably good to not perpetuate the error.
4. **Introduction**, *“Here, we describe a novel methodology to co-amplify all four EGFR exons 18–21 along with KRAS exon 2 in a single multiplex PCR”.*
  It's more like a novel application of a well described methodology supported by several previous references. The novelty, albeit rather incremental, is in combining exons from two different genes, using previously described approach. Should be stated as such.
5. The big appeal of the study is that it affords use of a minimal amount of FFPE sample. Please specify the amount of FFPE material used and yield of DNA to convey an idea of how little/much sample is needed to carry out this analysis.
6. The important consideration of sensitivity has not been addressed. This could be easily tested by assaying a serial dilution of known mutated cell line/FFPE DNA spiked in a wild-type background sample. This will add value to the study.
7. Addition of KRAS codon 61 should be considered as well. Or the difficulty in scaling up should be discussed. How difficult is it to add additional exons.
8. The concatenated PCR product may be amenable to Pyrosequencing to improve sensitivity of detection (particularly in case of low tumor content, low clonality of mutations as is expected in case of dynamically evolving tumors). This should be attempted/ discussed.
9. A direct comparison with the standard technique(s) currently used to test these mutations- in terms of amount of starting material needed, sensitivity of detection, time, and cost will help the argument of the new approach as a superior option.  

I have read this submission. I believe that I have an appropriate level of expertise to confirm that it is of an acceptable scientific standard, however I have significant reservations, as outlined above.

# Response

**Published:** 2015-07-31

**Authors:** Amit Dutt

**Affiliations:** ACTREC, Tata Memorial Center,, India

We thank the reviewer for describing the study as “a well conducted proof of principle report”, and for sharing elaborate comments and suggestions that has significantly improved the quality of the manuscript. Our response to specific concerns are as follows:

1. **Referee’s comments:** *Introduction, “These studies have direct implications for carrying out routine KRAS molecular testing along with EGFR mutations for precluding a patient with NSCLC from therapy with EGFR inhibitors, as approved for colorectal cancer (Li*è *vre et al., 2006).” Can the authors cite any reference wherein NSCLC patients are precluded from EGFR inhibitor therapy if they harbor KRAS mutations. The reference cited here is specific to colorectal cancer.*
**Author’s response:** *EGFR* and *KRAS* mutations occur mutually exclusive in NSCLC, which suggests functional redundancy. However, they predict contrasting response rate to tyrosine-kinase inhibitors (TKI). While *EGFR* mutation predicts longer progression-free survival rate, adverse prognosis is associated with patients harboring *KRAS* mutations. Thus, the recently reported co-occurrence of *KRAS* and *EGFR* activating mutations in 30 of 5125 patients, along with our study of co-occurrence of *KRAS* and *EGFR* activating mutations in 3 of 86 patients, raises a clinical concern about the relative value of *EGFR* and *KRAS* mutation status as predictors of outcome in NSCLC. As the reviewer may agree these studies may have obvious implications for routine *KRAS* testing in this disease, potentially precluding EGFR TKI therapy from some patients, similar to current practice in colorectal cancer.
  In principle, we fully agree with the reviewer that no direct evidence exist to preclude EGFR inhibitor therapy among patients co-harboring *EGFR *and *KRAS* mutation. In accordance with the reviewer’s suggestion we have revised the text to reflect the speculative implication of our methodology in NSCLC. Our modified text reads as follows:
“….While no evidence exists as yet, these studies may have implications for carrying out routine KRAS molecular testing along with EGFR mutations for precluding a patient with NSCLC from therapy with EGFR inhibitors, as approved for colorectal cancer (Liè vre et al., 2006)….”
2. **Referee’s comments:** *Introduction* *, “Of these direct sequencing is the most commonly used method worldwide”. Is this a personal opinion or there is a reference to support this. Should be cited.*
**Author’s response: **We thank the reviewer for pointing the omission. A relevant citation has been added. However, as our citation in manuscript is likely to be incomplete to summarize the field, some additional studies are mentioned below:
  Yatabe, Yasushi, et al. "EGFR Mutation Testing Practices within the Asia Pacific Region: Results of a Multicenter Diagnostic Survey." *Journal of Thoracic Oncology* 10.3 (2015): 438;
  Angulo, Bárbara, et al. "A comparison of EGFR mutation testing methods in lung carcinoma: direct sequencing, real-time PCR and immunohistochemistry." *PLoS One* 7.8 (2012): e43842;
  Ellison, Gillian, et al. "EGFR mutation testing in lung cancer: a review of available methods and their use for analysis of tumour tissue and cytology samples." *Journal of clinical pathology* 66.2 (2013): 79-89;
  Cappuzzo, Federico. "Methods for EGFR Mutation Testing." *Guide to Targeted Therapies: EGFR mutations in NSCLC*. Springer International Publishing, 2014. 19-24.
3. **Referee’s comments:** *Introduction* *, “concatenation or assembly of individually amplified exons from genomic DNA to generate a cDNA fragment has been described in earlier research”. cDNA specifically refers to complementary DNA derived from RNA through reverse transcriptase. Genomic PCR cannot be said to be used to generate cDNA fragment. I suspect this erroneous phrasing is picked up from a previous reference, but its probably good to not perpetuate the error.*
**Author’s response: **We agree and thank the reviewer for bringing it our attention. Our modified text reads as follows:
“….concatenation or assembly of individually amplified exons from genomic DNA to generate a coding fragment has been described in earlier research…”.
4. **Referee’s comments:** *Introduction* *, “Here, we describe a novel methodology to co-amplify all four EGFR exons 18–21 along with KRAS exon 2 in a single multiplex PCR”. It's more like a novel application of a well described methodology supported by several previous references. The novelty, albeit rather incremental, is in combining exons from two different genes, using previously described approach. Should be stated as such.*
**Author’s response: **As suggested by the reviewer, we have dropped the term “novel”. Our modified text reads as follows:
“….Here, we describe a methodology to co-amplify all four *EGFR* exons 18–21 along with *KRAS* exon 2 in a single multiplex PCR….”
5. **Referee’s comments:** *The big appeal of the study is that it affords use of a minimal amount of FFPE sample. Please specify the amount of FFPE material used and yield of DNA to convey an idea of how little/much sample is needed to carry out this analysis.*
**Author’s response: **As mentioned in the methodology section subtitled, “Multiplex PCR of *KRAS *exon 2 and *EGFR* exons 18-21”, multiplex PCR (50µl per reaction) was carried out in a single tube by using multiplex PCR kit (Qiagen) containing either 10 ng of genomic DNA from the NSCLC cell line or fresh frozen primary tumor, or 50 ng of genomic DNA from FFPE blocks. Furthermore, as mentioned under the methodology section subtitled, “Concatenation of exons and sequencing analysis”, 2 µl of multiplex PCR product was used as template in a 50 µl PCR reaction for concatenation.
6. **Referee’s comments:** *The important consideration of sensitivity has not been addressed. This could be easily tested by assaying a serial dilution of known mutated cell line/FFPE DNA spiked in a wild-type background sample. This will add value to the study.*
**Author’s response: **As mentioned under the last paragraph of the discussion section, “….As the limitation of the CRE strategy is defined by the sensitivity and resolution of the sequencing methodology adopted…” – which in this study has been Sanger Sequencing-- as the sensitivity of Sanger Sequencing is well established from FFPE and mutated cell line, we humbly differ from the reviewer that admixture experiment would add additional information.
7. **Referee’s comments:** *Addition of KRAS codon 61 should be considered as well. Or the difficulty in scaling up should be discussed. How difficult is it to add additional exons.*
**Author’s response:** *KRAS* codon 12 is mutated at a frequency of 25-50% in Caucasian population and 5-15% among East Asians. In a recent study we reported 18.6% *KRAS* codon 12 among Indian population (n=86). Given that *KRAS* codon 61 mutation exist at frequency < 1%; and, that none were found in our study in 86 patients, we decided to not include *KRAS* codon 61 mutation in this study to only present the proof of principle of the CRE methodology. However, we do agree with the reviewer about the significance of *KRAS* codon 61 mutation, and do hope to include it along with other known activating mutations in NSCLC.
  We submit that based on literature, additional exons can be added to the current methodology, as at least up to 10 genomic spliced exons fragment of 2295 bp has been described in literature using similar methodology.
8. **Referee’s comments:** *The concatenated PCR product may be amenable to Pyrosequencing to improve sensitivity of detection (particularly in case of low tumor content, low clonality of mutations as is expected in case of dynamically evolving tumors). This should be attempted/ discussed.*
**Author’s response: ** We fully agree with the reviewer’s insights that CRE can be utilized at high throughput mode to determine complete spectrum of *EGFR* and *KRAS *mutations using targeted next generation sequencing. Consistent with the reviewer’s suggestion the last paragraph of the discussion section reads, “… the limitation of the CRE strategy is defined by the sensitivity and resolution of the sequencing methodology adopted, concatenated *EGFR* and *KRAS* PCR products from multiple individuals—each tagged with unique bar code sequence—can be pooled and deep-sequenced using a NGS platform. The CRE strategy described here can reduce the labor and cost of performing individual PCR for all exons for each patient and effectively circumvent the noise due to variation in normalization for equimolar pooling of exons within the same sample at a resolution of single base.”
9. **Referee’s comments:** *A direct comparison with the standard technique(s) currently used to test these mutations- in terms of amount of starting material needed, sensitivity of detection, time, and cost will help the argument of the new approach as a superior option.  *
**Author’s response: ** As detailed in the manuscript, this proof of principle study introduces CRE as a methodology involving single multiplex-PCR followed by concatenation of the PCR product as one linear fragment for direct sequencing, as opposed to 5 rounds of PCR reaction followed by 10 rounds of sequencing reactions. A systematic comparative analysis is currently underway at our center using clinical cancer specimens for CRE compared to Sanger sequencing based methodology; SNaPShot PCR; Cobas system; Mass spec genotyping on a larger cohort sample, beyond the scope of this manuscript. Hence, we express our inability to include analysis from this ongoing study at this early on stage.

We sincerely thank the reviewer for the elaborate and detailed constructive review. Hope the reviewer will find the improved version of the manuscript acceptable for indexation.
